# Treatment of endometriosis: a review with comparison of 8 guidelines

**DOI:** 10.1186/s12905-021-01545-5

**Published:** 2021-11-29

**Authors:** Dimitrios Rafail Kalaitzopoulos, Nicolas Samartzis, Georgios N. Kolovos, Evangelia Mareti, Eleftherios Pierre Samartzis, Markus Eberhard, Kostantinos Dinas, Angelos Daniilidis

**Affiliations:** 1 Department of Gynecology and Obstetrics, Cantonal Hospital Schaffhausen, Geissbergstrasse 81, 8208 Schaffhausen, Switzerland; 2 Department of Obstetrics and Gynecology, Hippokratio Hospital, Aristotle University of Thessaloniki, Thessaloniki, Greece; 3 Department of Gynecology, University Hospital Zurich, Zurich, Switzerland

**Keywords:** Endometriosis, Pain, Infertility, Conservative treatment, Surgical treatment, Guidelines

## Abstract

**Background:**

Endometriosis, the presence of endometrial-like tissue outside the uterus, is a common clinical entity between women of reproductive age, with a prevalence of about 10%. Due to the variety of endometriosis-associated symptoms, a great variety of treatments have been implemented. The aim of this review is to give an overview on therapeutical approaches of eight national and international widely used guidelines.

**Methods:**

Six national (College National des Gynecologues et Obstetriciens Francais, National German Guideline (S2k), Society of Obstetricians and Gynaecologists of Canada, American College of Obstetricians (ACOG) and Gynecologists, American Society for Reproductive Medicine (ASRM) and National Institute for Health and Care (NICE) and two international (World Endometriosis Society, European Society of Human Reproduction and Embryology) guidelines are included in this review.

**Conclusion:**

All the above-mentioned guidelines agree that the combined oral contraceptive pill, progestogens are therapies recommended for endometriosis associated pain. Concerning infertility, there is no clear consensus about surgical treatment. Discrepancies are also found on recommendation of the second- and third-line treatments.

## Introduction

Endometriosis is an inflammatory entity with the presence of endometrial-like tissue outside the uterine cavity. It mainly affects women of reproductive age, with a prevalence of about 10% [1]. The wide range of symptoms, such as chronic pelvic pain, dysmenorrhea, infertility, dyspareunia, dysuria, dyschezia and fatigue which characterize this estrogen-dependent condition, result to a delayed diagnosis. The importance of endometriosis as a major health issue with socioeconomical impact is underlined by the EndoCost study, which showed that costs arising from endometriosis are comparable to that of other chronic diseases, such as diabetes mellitus [2]. Many gynecological societies have published different guidelines in order to help the clinicians with the diagnosis and treatment of endometriosis. The variety of the available treatments in combination with complexity of this illness leads to significant discrepancies between the recommendations. As previous showed, there is only a 7% agreement between the widely used guidelines and none of them is following the Appraisal of Guidelines for Research and Evaluation II (AGREE-II) protocol [3]. The aim of this review is to give an overview about treatment of endometriosis after comparing eight widely used endometriosis guidelines.

## Material and methods

This review includes six national and two international guidelines of endometriosis. Two independent reviewers (DRK, NS) selected all the included guidelines available by September 2020 and extracted all the recommendation in standardized excel sheets according the type of recommendation and its evidence grade (Tables 1, 2).

**Table 1:**
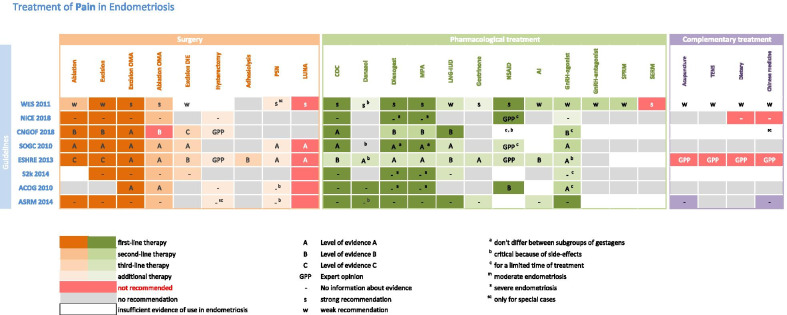
Treatment of Pain in Endometriosis

**Table 2:**
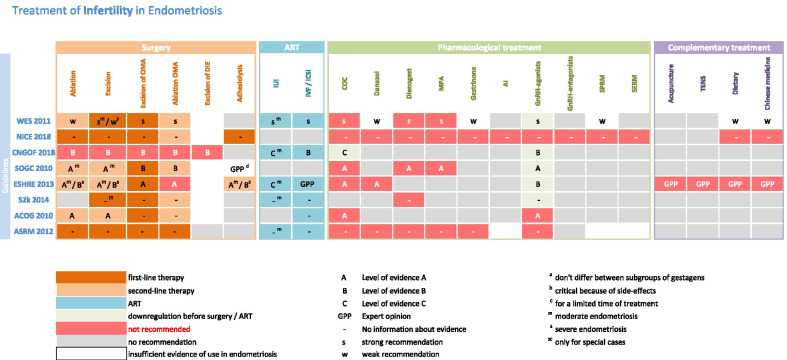
Treatment of Infertility in Endometriosis

National guidelines: College National des Gynecologues et Obstetriciens Francais 2018 (CNGOF) [4], National German Guideline (S2k) 2014 [5], Society of Obstetricians and Gynaecologists of Canada (SOGC) 2010 [6], American College of Obstetricians and Gynecologists (ACOG) 2010 [7], American Society for Reproductive Medicine (ASRM) 2012 for infertility and 2014 for endometriosis associated pain [8, 9] and National Institute for Health and Care (NICE) 2018 [10].

International guidelines: World Endometriosis Society (WES) 2011 [11] and European Society of Human Reproduction and Embryology (ESHRE) 2013 [12].

### Surgical treatment of endometriosis

#### Surgical approach

All the included guidelines recommend laparoscopic surgery in preference to laparotomy for chronic pain of endometriosis and infertility, because of less pain, shorter duration of hospitalisation, quicker recovery and better cosmetic result [13]. ESHRE guidelines reports that laparotomy and laparoscopy are equally effective in the treatment of endometriosis-associated pain. None of the above guidelines mentions robotic surgery as an option for endometriosis surgery. A meta-analysis of the available studies showed no other difference in perioperative outcomes between robotic and conventional laparoscopic surgery, except the longer time that is needed in robotic surgery [14]. ASRM suggests that multiple surgical procedures should be avoided because of adhesions and reduction of ovarian reserves. According to ESHRE, CNGOF guidelines no preoperative hormonal treatment is recommended, while the above guidelines and additional the NICE and SOGC guidelines recommend that postoperative hormonal treatment could be considered a secondary prevention. ASRM, ACOG, S2k and WES report conflicting evidence about postoperative treatment in women with endometriosis associated pain or endometrioma.

Vaginal procedures for treatment of deep infiltrating endometriosis are discussed in CNGOF 2007, S2k and ESHRE guidelines. CNGOF 2007 underlines that skilled surgeons should carry out laparoscopically assisted vaginal procedures and according to experience of the guideline development group exclusively vaginal operations are not recommended, while the latest version of CNGOF guideline does not take position on this issue. The available literature about laparoscopically assisted vaginal procedures includes a few retrospective studies with small number of patients, which conclude that this technic could be considered only for the treatment of rectovaginal endometriosis (15).

#### Peritoneal endometriosis

CNGOF, ESHRE, S2k, ASRM and SOGC recommend the treatment of superficial endometriosis in patients with endometriosis associated pain. CNGOF, ESHRE and ASRM do not give a preference about the different techniques (ablation, excision), while both S2k and SOGC explicitly mention the lack of evidence. WES recommends in general excision of any kind of endometriosis lesions. The recent evidence is though unclear about the benefit of peritoneal endometriosis excision in women with chronic pain [16, 17].

CNGOF, NICE, WES, ESHREM S2k ACOG and ASRM recommendations agree that women with suspected mild endometriosis and infertility should be considered candidates for surgical treatment. A Cochrane meta-analyses showed an increased birth rate odds ratio of 1.94, 95% CI 1.20–3.16 for patients with infertility who underwent surgery for excision of the endometrial implants [18].

#### Ovarian endometriosis

For surgical treatment all of the included guidelines follow the recommendation of Hart et al. [19]. In this Cochrane review the authors concluded that laparoscopically cystectomy of endometriomas measuring more than 3 cm was superior to drainage and ablation with electrocoagulation in terms of lower recurrence of dysmenorrhoea, dyspareunia, cyst recurrences and the need for further surgical interventions. Only ESHRE and CNGOF discuss laser vaporization in treatment of endometriomas. After “one-step” laser vaporization, a greater recurrence of endometriosis related ovarian cysts was observed after 12 months of follow up according to Carmona et al. [20]. Nevertheless, recurrence rate did not differ statistically significant after 5 years compared to cystectomy. According to ASRM, simple drainage has a little therapeutic value as recurrence rate is 80–100% and therefore is no longer being performed. ACOG points out, that, cyst wall should be removed to obtain a histological sample, especially in women without a previous diagnosis of endometriosis, in order to exclude the small risk of malignancy [21].

CNGOF and ASRM underline that surgical treatment of endometriomas by cystectomy or ablation can reduce ovarian reserve, with negative implication on fertility. The risk increases for woman with large, recurrent or bilateral endometriomas. Measurement of AMH prior to ovarian surgery has to be considered.

In infertile patients surgery of endometriomas does not improve the outcome of IVF according to the same guideline. ESHRE recommends that cystectomy possible should be performed, rather than ablation or other therapeutic managements in infertile patients, while according to S2k, the effect of ovarian endometriomas on the outcome of IVF is unclear. WES reminds that oocyte freezing should be discussed in young women prior to surgery of endometrioma [22]. Conclusively independent of the surgical method of intervention, the concept of minimizing the negative effects on the ovarian reserve should consist a priority [23].

#### Deep infiltrating endometriosis (DIE)

All guidelines except of NICE and ASRM, recommend excision of deep infiltrating endometriosis nodules for endometriosis associated pain. The management about fertility is controversial. This procedure because of the complexity should be performed by experts. ESHRE mentions that surgery in women with deep endometriosis is associated with substantial intraoperative and postoperative complication rates and according to CNGOF possible complications are leaks from anastomosis, fistulas, rectal dysfunction and bladder atony caused by surgical alteration of the hypogastric plexus (splanchnic nerves) which are unavoidable in some cases. ESHRE underlines the controversial results between shaving and segmental resection. Bladder endometriosis excision is recommended by ESHRE and CNGOF. Last but not least, NICE and the German group recommend a preoperative imaging with ultrasound or MRI. ESGE/ESHRE/WES published on February 2020 recommendations on the technical aspect of different surgical approaches for deep infiltrating endometriosis [24].

#### Hysterectomy

All of the above-mentioned societies concur that hysterectomy with the simultaneous excision of endometriotic lesions is considered to be the last solution in women who have fulfilled their family planning and fail to respond to more conservative treatments. When hysterectomy is going to be performed, according to WES and NICE guidelines, it should be done laparoscopically. As far as ovarian preservation is concerned some discrepancies between the recommendations of the above guidelines exist. According to CNGOF, NICE, ESHRE and ASRM hysterectomy with bilateral salpingo-oophorectomy (TAHBSO) should be the preferred in the prospect of lowering the risk pain recurrence and reoperation, while JOGC and ACOG refer that ovarian preservation should be considered in patients with normal ovaries. If HRT needed for the treatment of menopausal symptoms, the German society (S2k) and ASRM recommend the use of combined estrogen-progestogen therapy. The risk of endometriosis recurrence after hysterectomy constantly exists and diverse theories have been proposed, such as residual microscopic foci, hormonal factors, ovarian remnants, uterine morcellation, lymphovascular invasion, and de-novo disease [25].

According to Vercellini et al., patients should always be informed that there is an approximate 15% probability of pain persistence after standard hysterectomy with a 3–5% risk of worsening of pain or development of new symptoms [26].

A tailored radical hysterectomy for patients with deep infiltrating endometriosis including removal of the uterus, adnexa, posterior and anterior parametria, endometriotic lesions and upper one-third of the vagina with lesions of lateral and posterior vaginal epithelium is proposed by Fedele et al. [27].

#### Adhesiolysis

Adhesions have a negative influence on fertility by altering the adnexal anatomy, gamete and embryo transport [28]. Although there is insufficient data about the effect of isolated adhesiolysis on endometriosis associated pain, only two societies ESHRE reports adhesiolysis as a method for treating endometriosis-associated pain and recommend that clinicians should be aware of use of anti-adhesion agents in order to prevent and minimize adhesion formation. With regards to fertility, ESHRE and NICE recommend that adhesiolysis improves the chance of spontaneous pregnancy, while according to JOGC anti-adhesion agents may reduce the formation of adhesions but the outcome in fertility is not proven.

Conclusively, a Cochrane review reports no evidence of available agents, oxidised regenerated cellulose (Interceed ®), expanded polytetrafluoroethylene (Gore-Tex ®) and sodium hyaluronate with carboxymethylcellulose (Seprafilm ®) on pelvic pain and fertility [29].

#### Laparoscopic uterine nerve ablation (LUNA) and presacral neurectomy (PSN)

Many societies including the WES, S2k, ASRM and ESHRE have examined the possible role of laparoscopic uterine nerve ablation for the management of endometriosis associated pain. Laparoscopic uterine nerve ablation (LUNA) is a technique designed to disrupt the efferent nerve fibres in the uterosacral ligaments with the purpose of decreasing uterine pain in women with intractable dysmenorrhea. Ultimately, a common conclusion is reached according to Cochrane Review of Proctor et al., which showed that LUNA has no beneficial effect on dysmenorrhea and endometriosis-associated chronic pain [30].

On the other hand, presacral neurectomy has been suggested as an effective additional method for treatment of midline pain in patient with endometriosis [31]. Although it is important to recognize that presacral neurectomy is a technically challenging procedure associated with significant risk of bleeding from the adjacent venous plexus. Possible side effects of presacral neurectomy such as haematoma, constipation and urinary dysfunction are mentioned by the ACOG guidelines. Consequently, ESHRE guideline emphasizes that presacral neurectomy requires a high degree of skill from an experienced surgical team.

### Medical treatment of endometriosis

#### Progestines

All eight guidelines recommend progestins as first-line medical treatment for pain in endometriosis. In this paragraph we try to investigate the different types and ways of administration of progestins on the treatment of endometriosis.

##### Dienogest

Dienogest (DNG) is 19-nortestosterone derivative, a fourth generation orally active progestogen with a high specificity for the progesterone receptor (PR) [32]. The most common used dosage, 2 mg per day, causes only a minimal reduction of the estrogen levels, thus, none hypoestrogenic side effect is described [33]. WES and S2k recommend dienogest prior to other progestines. S2k and CNGOF underline, that in two RCTs the administration of dienogest showed comparable efficacy to GnRH-analogues with better tolerability [34, 35].

##### Medroxyprogesterone acetate

Medroxyprogesterone acetate, a 17OH-progesterone derivative, is commonly used as a three monthly intramuscularly or subcutaneously administered contraceptive method (58). It belongs to first line therapies for endometriosis-associated pain according to the two American societies, the Canadian society and ESHRE. The evidence grade varies between the above societies. WES underlines the weak evidence grade and classifies the above therapy as a second line treatment.

Medroxyprogesterone acetate seems to be an effective and very economical therapy in relieving endometriosis-associated pain, with substantially less bone loss than GnRH agonists [36].

##### Levonorgestrel-IUS

LNG-IUS is a commonly used mechanic and hormonal contraceptive method, releasing a 19-nortesterone derivative directly into the uterine cavity over a period of 5 years. The proposed mechanisms of levonorgestrel-IUS on endometriosis therapy are the induction of endometrial glandular atrophy, transformation of the stroma, the downregulation of endometrial cell proliferation and the intensification in apoptotic activities [37].

ACOG mentions, that levonorgestrel intrauterine system is similar effective as GnRH agonist in reducing endometriosis-associated pelvic. ASRM and CNGOF in concordance to a recent meta-analysis point out, that levonorgestrel intrauterine system reduces the recurrence of dysmenorrhoea after surgical treatment of endometriosis [38].

#### Combined Oral Contraceptives Pills

The combined oral contraceptive pill (COC) is a widespread contraceptive method which is also used widely from clinicians empirically in patients with dysmenorrhea. Most of the included guidelines propose combined oral contraceptives as a first empirical medical treatment in endometriosis associated pain before performing diagnostic laparoscopy although the reported level of evidence differs. A meta-analysis from Brown et al. showed that use of COCs in comparison with placebo is associated with relief of dysmenorrhoea, however in comparison with GnRH analogue there were no superiority of treatment. The authors of this meta-analysis underline that the above conclusions should not be generalised, because of the limited available evidence [39].

Only CNGOF and WES refer the possibility of using COCs for downregulation before ART in patients with endometriosis. A Cochrane review underlines the limitation of the available data on the role of COC before IVF [40].

#### NSAIDs

Nonsteroidal anti-inflammatory drugs (NSAIDs) are widely used for symptomatic treatment of dysmenorrhoea and acyclic pelvic pain. In most of the guidelines of these eight societies the use of NSAIDs is described. NSAIDs are considered to be a symptomatic first line treatment, a long-term use is not recommended because of the possible side effects. The last Cochrane review, showed lack of high quality evidence and no difference in pain relief in comparison with placebo [41].

#### Gonadotropin Releasing Hormones (GnRH) agonists

GnRH agonists use in endometriosis patients is reserved for patients with persistent symptoms after the use of first line therapy. All the above societies agree that GnRH agonists can reduce the endometriosis associated pain. CNGOF recommends that GnRH agonists with add-back therapy should not be used for more than one year, SOGC recommends a duration not longer than 6 months, while ESHRE underlines that there is not sufficient evidence about the duration of the above therapy. German society (S2k) recommends that GnRH agonists are not appropriate treatment for ovarian endometriomas[42]. In infertile patients is concerned, WES, CNGOF and SOGC propose downregulation with GnRH agonist 3–6 months prior to IVF in order to improve pregnancy rate. ACOG and German society contradict to this recommendation because clinical pregnancy rate and live birth rate data is not conclusive [43].

The clinicians have to take into consideration the hypoestrogenic side effects for example vasomotor symptoms and accelerated bone loss. According to FDA the use of the above medicaments should be restricted to six months. Progestin-only and progestin with low dose oestrogen (0.625 mg) add-back therapy are both associated with reduction of the side effects [44].

#### Gonadotropin Releasing Hormones (GnRH) antagonists

GnRH antagonist is new promising medical treatments for women with endometriosis associated pain, inducing a dose-dependent ovarian suppression. Two RCTs compared elagolix® with placebo and showed a reduction of dysmenorrhoea and nonmestrual pelvic pain, although the comparison between elagolix® and medroxyprogesterone acetate did not show significant difference in pain reduction. Studies comparing the above medication with other possible treatment and long term outcomes are not published yet [45]. Only four guidelines (ESHRE, ASRM, CNGOF and WES) refer GnRH antagonist as a possible therapeutic option for endometriosis related pain although all of the above underline that the evidence is not enough.

#### Aromatase inhibitors

Aromatase inhibitors block the enzymatic activity of aromatase reducing the synthesis of estrogen in the ovaries and peripheral tissues [46].

NICE and German society (S2k) guidelines do not refer aromatase inhibitors as a possible endometriosis treatment. All the other societies agree that it could be a second line therapy for endometriosis-associated pain reduction, although the evidence is not enough. ASRM guidelines statement is that the above therapy should not be considered as a definitive therapy, as it is not FDA approved for endometriosis. ESHRE underlines that aromatase inhibitors could be used in combination with oral contraceptive pills, progestagens, or GnRH analogues in order to avoid the ovarian stimulation.

Aromatase inhibitors reduce endometriosis-associated pain, intestinal symptoms, urinary symptoms and decrease the volume of laparoscopically visible endometriosis, rectovaginal infiltrating endometriosis and endometriomas. The above treatment improve the quality of life when used with gestagens, oral contraceptives or GnRH-agonists. Ferrero et al. conclude that aromatase inhibitors should be offered to women with pain persistence after previous surgical and hormonal treatment [47].

Endometriosis can affect about 2–5% of postmenopausal patients. In this group, aromatase inhibitors seem to be a possible medical treatment as the largest amount of estrogens is produced from extra-ovarian sources [48]. Long-term use is associated with hypoestrogenic side effects, such as vaginal dryness, hot flushes, headache, arthralgia and with an increased risk for bone fractures, osteoporosis and osteopenia [46].

#### Danazol

Danazol is an androgenic drug, that was used for the treatment of endometriosis related pain for more than 40 years. Because of the hyperandrogenic side effects (weight gain, acne, hirsutisms, breast atrophy and virilisation), low dose vaginal administration has been proposed [49].

As far as the use of danazol in treatment of endometriosis pain is concerned, ACOG is the only guideline which still propose the above medication as a possible first line therapy. WES, ESHRE and SOGC are critical because of the side effects and WES recommends to use it only in women who have already had a well-tolerated treatment with danazol before. S2k and ASRM do not have an official recommendation.

According to ESHRE and NICE guidelines, danazol for infertile patients should not be recommended while according to WES there is not enough evidence.

#### Gestrinone

Gestrinone, one of the first drugs for the treatment of endometriosis and myomas, which acts centrally on the hypothalamic pituitary system by supressing the release of lutenizing hormone (LH) and follicle-stimulating hormone (FSH) is actually not widely used [50]. Only WES and ESHRE discuss the use of gestrinone as a possible medication for the treatment of endometriosis related pain, while ASRM underlines that this therapy is at the moment not available in the United States. WES is the only society which refers that there is limited evidence about the role of gestrinone in the therapy of women with infertility.

#### Selective estrogen receptor modulators (SERM)

SERMs have tissue-specific estrogen receptor agonist and antagonist effects. ESHRE, CNGOF and ASRM refer that there is not enough evidence for treatment of endometriosis associated pain. NICE guideline does not recommend SERM as endometriosis related infertility treatment.

##### Selective progesterone receptor modulators (SPRM)

Selective progesterone receptor modulators have a variable effect on progesterone receptors which varies from pure agonistic to pure antagonistic. A systematic review showed that mifepristone, is more effective than placebo for dysmenorrhoea and dyspareunia, although the current literature does not provide enough evidence for long-term safety and efficacy of this treatment [51].

WES recommends that SPRM could be a second line therapy, while ESHRE, CNGOF and ASRM underlines that evidence is not sufficient. NICE guideline does not recommend SPRM as a treatment for endometriosis-associated infertility.

##### Nonhormone treatments

A possible option for endometriosis-associated pain is pentoxifylline, a nonselective phosphodiesterase inhibitor with immunomodulatory properties, which, according to WES, ESHRE and ASRM, could not be recommended as a standard therapy for endometriosis due to the lack of evidence to date [52].

In the same way, antiangiogenic agents, such as anti-TNF-a and infliximab have been evaluated as a potential endometriosis treatment. WES, CNGOF and ASRM agree that there is no benefit according to available studies [53].

### Complementary therapies

#### Acupuncture

Acupuncture nowadays serves as complementary method for the treatment of endometriosis related pain when conservative and surgical methods fail to relieve the patient’s symptoms, reducing the pain through the mechanism of stimulation by diffusion of noxious inhibitory controls. A meta-analysis including two RCTs comparing acupuncture with placebo showed a significant reduction of pelvic pain (RR –1.93, 95% CI –3.33, –0.53, *p* = 0.007) [54]. According to CNGOF and ASRM acupuncture can improve the quality of life in patient with endometriosis-related pain. SOGC recommends that acupuncture should be conducted by specialists. On the other side WES, S2k, ESHRE and NICE does not recommend acupuncture as therapy for endometriosis associated symptoms.

#### Electrotherapy

Transcutaneous Electrical Nerve Stimulation (TENS) is the most commonly used electrical stimulation for pain therapy by directly blocking transmission of pain signals along nerves. After comparing acupuncture with TENS applied in the sacral region by endometriosis patients, both methods showed reduction of chronic pelvic pain (*p* < 0.001), deep dyspareunia (*p* = 0.001) and improvement of the quality of life (*p* < 0.001) of women with deep endometriosis [54]. CNGOF reports, that TENS has been found to be of value in primary dysmenorrhoea. WES and S2k agree that the evidence is not sufficient, while ESHRE recommends the above methods to be used only by experts and SOGC does not recommend this complementary treatment.

#### Dietary products and vitamins

Limited evidence exists in the bibliography on the effectiveness of the dietary methods to face the endometriosis related pain. Crucial part of this category is the dietary supplements and vitamins, especially B1, B6 and D [55–57]. Although a lot of medications and interventional methods have been studied, there is a lack of a randomized controlled trial to examine the efficacy of the behavioural and lifestyle habits. As a conclusion, these methods have not yet been established and are not even mentioned by most of the guidelines as an effective method to reduce the pain induced by endometriosis lesions.

## Conclusion

Conclusively, this is a review and summary of the eight most widely accepted guidelines concerning the management of endometriosis. Pain and infertility are the major components of endometriosis that most usually lead patients in seeking an expert opinion.

Regarding pharmacological therapies of endometriosis associated pain, the most of the included guidelines suggest progestins, either in the form of dienogest or of medroxyprogestetrone acetate, and combined oral contraceptives as first line therapy with a great evidence grade. GNRH-agonists and levonorgestrel intrauterine system could be considered as second line treatment. About the remaining medical options such as danazol, gestrinone, aromatase inhibitors, SERMs and SPRMs because of the limited evidence there are discrepancies between the guidelines. Important is also the role of surgery on treatment endometriosis related pain, where the standard of practice is the excision of the endometrial implants as well as the excision of the endometriomas. In general, it is advised to handle the ovarian tissue as atraumatic as possible, to reduce the decrease of ovarian reserve. At last, complementary options such as dietary products, acupuncture and electrotherapy are not yet studied enough in order to have a better perspective of their role.

When it comes to infertility the available therapeutic options and the therapeutic strategies differ in comparison to the endometriosis associated pain. The surgical procedures such as excision of endometriomas and endometriosis excision have gained the greatest evidence grade and consist the standard approach. Ablation of the ovarian endometriosis is a second line therapy, while pharmacological therapies are in principle not recommended, except GNRH-agonists that can be used as a downregulation therapy before IVF or surgery. Likewise with pain, complementary therapies are not yet considered as a therapeutic option because of the lack of evidence.

## Data Availability

Data sharing is not applicable to this article as no datasets were generated or analysed during the current study.

## Abbreviations

CNGOF: College National des Gynecologues et Obstetriciens Francais
S2k: National German Guideline
SOGC: Society of Obstetricians and Gynaecologists of Canada
AGOC: American College of Obstetricians and Gynecologists
ASRM: American Society for Reproductive Medicine
NICE: National Institute for Health and Care Excellence
WES: World Endometriosis Society
ESHRE: European Society of Human Reproduction and Embryology
IUS: Intrauterine system
GnRH: Gonadotropin-releasing hormone
SERM: Selective estrogen receptor modulators
SPRM: Selective progesterone receptor modulators
RCT: Randomized control trial
NSAID: Nonsteroidal anti-inflammatory drugs
HRT: Hormone replacement therapy
COCP: Combined oral contraceptive pill
LUNA: Laparoscopic uterine nerve ablation
PSN: Presacral neurectomy
DIE: Deep infiltrating endometriosis
FSH: Follicle-stimulating hormone
LH: Luteinizing hormone
TENS: Transcutaneous Electrical Nerve Stimulation
